# Tick-borne pathogens in dogs and their ticks in France: Molecular and serological evidence from a multicenter participatory study

**DOI:** 10.1016/j.onehlt.2026.101487

**Published:** 2026-06-17

**Authors:** Djamel Tahir, Sophie Dupuis, Virginie Geolier-Lapeyronie, Ambre Sibari, Yousra El-Ouatik, Cécile Collignon, Samantha Favy, Thomas Blondel, Alessia Crippa, Laurence Malandrin, Elisabeth Ferquel, Marie Varloud, Valérie Choumet

**Affiliations:** a Institut Pasteur, Environnement et Risques Infectieux, Université Paris Cité, 75015 Paris, France; b Ceva Santé Animale, 10 Avenue de la Ballastière, 33500 Libourne, France; c Mon Véto, 101 Boulevard de l'Europe, 76000 Rouen, France; d Oniris, INRAE, BIOEPAR, 44300 Nantes, France

**Keywords:** One health, Tick-borne pathogens, Vector-borne disease, Lyme disease, Anaplasmosis, Piroplasmosis, Ehrlichiosis, Canine, Molecular surveillance, Participatory approach

## Abstract

Canine tick-borne diseases (TBDs) are expanding globally, representing an increasing concern for both veterinary and public health. Dogs, due to their close contact with humans and frequent exposure to ticks, may serve as valuable sentinels for zoonotic risk. Between December 2022 and December 2023, we conducted a year-long multicenter participatory pilot survey in France involving veterinary clinics, dog owners, and research laboratories. Ticks and blood samples were collected from 82 dogs presented in 41 veterinary practices across 34 departments. Tick species were identified morphologically, and genomic DNA extracted from ticks and canine blood and/or serum samples was screened for selected tick-borne pathogens (TBPs) using PCR and sequencing. Serological analyses were also performed. A total of 165 ticks were collected from enrolled dogs, including *Dermacentor reticulatus* (29.7%), *Ixodes ricinus* (18.2%), *Rhipicephalus sanguineus* s.l. (16.4%), *I. hexagonus* (6.7%), and *D. marginatus* (0.6%). Tick submissions were recorded throughout the study period, with temporal variations observed among tick genera. Molecular screening identified several TBPs in dogs, including *Borrelia garinii* (*n* = 5) and *Babesia canis canis* (n = 5). Serological analyses revealed antibodies against *Ehrlichia* spp. and *Anaplasma* spp. in one and three dogs, respectively. Several infected dogs were asymptomatic. In ticks, the main TBPs detected included *B. garinii*, *B. canis canis*, *Rickettsia massiliae*, and *R. raoultii*, as well as several endosymbionts. This multicenter participatory pilot study supports the feasibility of a One Health surveillance approach based on veterinary networks for monitoring tick species and TBPs. Although the study was not designed to assess national prevalence or validate dogs as sentinels through comparison with human surveillance data, the findings provide proof-of-concept for the potential value of integrating veterinary and public health surveillance systems to improve our understanding of TBP circulation in France.

## Highlights

• Multiple tick species were collected from dogs in a multicenter participatory study conducted in France.
• Tick-borne pathogens, including *Borrelia garinii* and *Babesia canis canis*, were detected in dogs.
• Several zoonotic tick-borne microorganisms, including *Borrelia garinii*, *Rickettsia massiliae*, *Rickettsia raoultii*, and *Babesia venatorum*, were detected.
• The study provides proof-of-concept for a veterinary network-based participatory surveillance system for tick-borne pathogens.

## Graphical abstract

**Figure:**
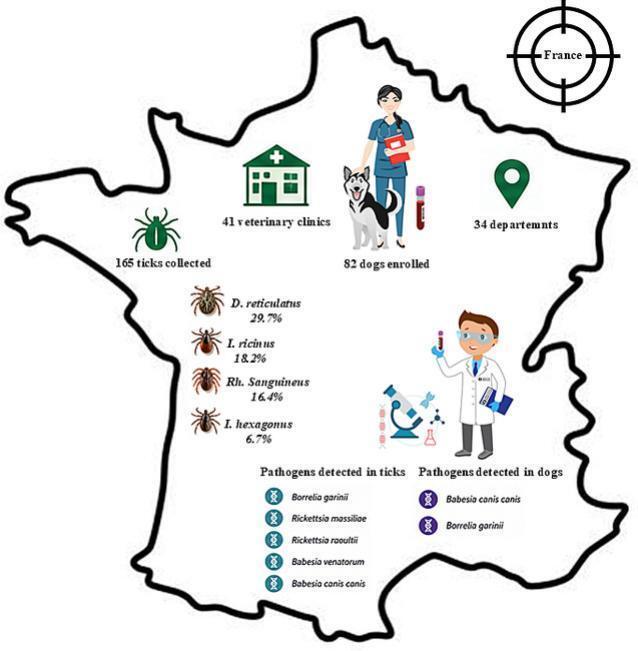

## 1 Introduction

Ticks are obligate hematophagous ectoparasites of the order Ixodida and are among the most important vectors of pathogens affecting both animals and humans worldwide [1], [2], [3]. Through their capacity to transmit a wide diversity of infectious agents, including bacteria, protozoa, and viruses, ticks play a central role in the epidemiology of vector-borne diseases (VBDs) and represent a growing concern for global health [1], [2], [3], [4]. In Europe, the incidence and geographic distribution of tick-borne diseases (TBDs) have increased markedly in recent decades, driven by environmental changes, host dynamics, and human activities [5], [6].

These evolving epidemiological patterns highlight the need for integrated surveillance systems capable of capturing pathogen circulation across the human–animal–environment interface. However, surveillance of TBDs remains largely fragmented, often relying on either human clinical data or targeted entomological studies, with limited integration of veterinary data. This lack of coordination may delay the detection of emerging risks and hinder the implementation of effective prevention strategies. In response to these challenges, several European initiatives have promoted One Health approaches to VBD surveillance. Notably, the VectorNet project, jointly coordinated by the European Centre for Disease Prevention and Control (ECDC) and the European Food Safety Authority (EFSA), supports the harmonized collection and analysis of data on vectors and VBPs across Europe [7], [8]. More broadly, European surveillance initiatives increasingly promote the integration of veterinary, environmental, entomological, and public health data within a One Health framework to improve the detection of emerging vector-borne threats [9], [10].

Domestic dogs represent a particularly relevant interface host in this context. In Western Europe, dogs are commonly infested by *Ixodes ricinus*, *Dermacentor reticulatus*, *Rhipicephalus sanguineus* sensu lato, and *Ixodes hexagonus* [11], [12], [13], [14]. These tick species are known vectors of numerous pathogens of veterinary and zoonotic importance, including agents of anaplasmosis, babesiosis, ehrlichiosis, Lyme borreliosis, and tick-borne encephalitis [15], [16], [17], [18].

Beyond their role as hosts, dogs are increasingly recognized as sentinels for environmental and zoonotic hazards. Their frequent exposure to tick-infested environments, combined with their close proximity to humans, places them at the intersection of animal and human health [19], [20], [21]. Monitoring TBPs in dogs may therefore provide indirect but valuable information on pathogen circulation and potential human exposure risk within shared ecosystems.

Despite this potential, nationwide integrated surveillance systems leveraging veterinary networks remain underdeveloped in many European countries. In France, although several studies have reported the presence of ticks and associated pathogens in animals, large-scale, participatory approaches integrating multiple stakeholders, including veterinarians, pet owners, research institutions, and private partners, are still scarce.

**Fig. 1:**
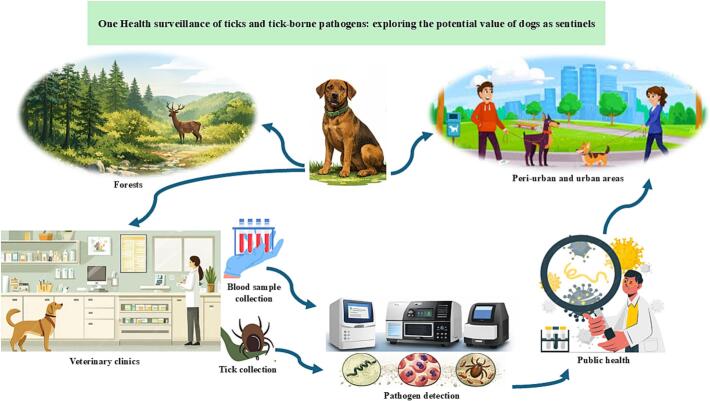
Conceptual overview of a One Health surveillance system for tick-borne pathogens in dogs.

In this context, the present study aimed to implement a multicenter participatory surveillance system to investigate tick species and associated TBPs in dogs from participating veterinary clinics across France (Fig. 1). By combining standardized sampling through a veterinary network, molecular pathogen detection, and clinical data collection, this pilot study provides preliminary information on the occurrence of TBPs in dogs and their associated ticks. More broadly, it provides proof-of-concept for the feasibility and potential value of a One Health surveillance approach based on veterinary networks to support the development of coordinated surveillance strategies for tick-borne diseases.

## 2 Materials and methods

### 2.1 Ethical statement and sample collection

The survey was conducted from December 2022 to December 2023, involving 41 veterinary practices (MonVeto network) across France. Dog owners were informed about the Collec'Tiques study by their veterinarians.

Tick specimens were collected from each dog presented for veterinary examination. In addition, four milliliters (4 mL) of blood were drawn from the cephalic vein into plain or EDTA-containing vacutainer tubes. Blood samples were obtained specifically for the purposes of the study and were not collected as part of routine veterinary diagnostic procedures. Only dogs with confirmed tick infestations were enrolled. All experimental protocols in this study were ethically approved by the Avogadro LS Ethics Committee (062CE-CEEA, France) under the notification number 2022–00928. Written consent was obtained from all dog owners. Data collected included the geographical location of the clinic and animal's sex. Veterinarians completed clinical case forms documenting clinical signs, history, and potential tick-borne infection related symptoms.

Participating veterinary clinics were supplied with standardized sample collection kits free of charge. Each kit contained a SNAP 4Dx Plus test (IDEXX Laboratories), blood collection tubes, a standardized data collection form, and pre-stamped envelopes to ensure proper packaging and shipment of samples to the Institut Pasteur in Paris. Blood and/or serum samples were shipped at room temperature and delivered to the laboratory within 24 h of collection. Upon receipt, samples were stored at −20 °C until DNA extraction.

To ensure consistency across study sites, all participating clinics followed a standardized sampling protocol. Veterinarians received detailed written instructions describing the inclusion criteria, tick collection procedures and blood sampling. In addition, a demonstration video outlining the study workflow and sampling procedures was made available through the Mon Véto intranet platform prior to study initiation.

Notably, once laboratory analyses were validated, results were systematically transmitted to the corresponding veterinary clinics on an ongoing basis. This real-time reporting ensured prompt clinical interpretation and facilitated appropriate case management and follow-up when necessary.

### 2.2 Serological screening and microscopic Babesia detection in peripheral blood

Blood samples were analyzed at the clinic for antibodies to *A. phagocytophilum*/*A. platys*, *B. burgdorferi* s.l., *E. canis*/*E. ewingii*, and for *Dirofilaria immitis* antigen using the SNAP 4Dx Plus point-of-care assay according to the manufacturer's instructions. In addition, Giemsa-stained blood smears prepared by the practitioners were examined at 1000 × magnification for the presence of *Babesia*-like intraerythrocytic piroplasms. Occasional extracellular forms, likely resulting from erythrocyte rupture during smear preparation, were also recorded.

### 2.3 Tick identification

Ticks were collected into aerated tubes containing water-moistened cotton and transported to the Institut Pasteur in Paris for further analysis. Each specimen was categorized by sex and developmental stage and morphologically identified to the species level using established taxonomic keys [22], [23]. Damaged specimens were identified to genus level only.

### 2.4 Molecular detection and genetic characterization of pathogens

Genomic DNA was extracted from 200 μL of each blood or serum sample using the DNeasy Blood and Tissue Kit (QIAGEN GmbH, Hilden, Germany), following the manufacturer's instructions, with a final elution volume of 100 μL. Extraction blanks were included as negative controls during each extraction batch.

Ticks were rinsed with distilled water and dried on sterile filter paper. Each specimen was sectioned into small pieces using sterile scalpels and homogenized with the Precellys Evolution Touch homogenizer (Bertin Technologies, France) in 300 μL of DNA extraction buffer and centrifuged at 10000 rpm for 40 s. Subsequently, 20 μL of proteinase K was added, and samples were incubated overnight at 37 °C. Genomic DNA was then extracted according to the manufacturer's instructions using DNeasy Blood and Tissue Kit (QIAGEN GmbH, Hilden, Germany) and stored at −20 °C until further use.

Target sequences of *Anaplasma*/*Ehrlichia* spp., *Babesia* spp. and *Rickettsia* spp. were detected by standard PCR assays (sPCR), as previously described (Supplementary Table 1). The universal 16S rRNA primers (EHR521 and EHR747, as well as EHR16SD and EHR16SR) used for the detection of *Anaplasma*, *Ehrlichia* and *Rickettsia* also amplified DNA from certain bacterial endosymbionts, which were subsequently identified by sequencing analysis. In addition, the presence of *Babesia* spp. and *B. burgdorferi* s.l. DNA was assessed using nested PCR (nPCR) (Supplementary Table 1).

To minimize the risk of contamination, pre- and post-PCR procedures were conducted in physically separated laboratory areas. Positive controls, no-template negative controls, and extraction blanks were included in each PCR run. All positive samples were confirmed by repeat amplification and sequencing.

### 2.5 Sequencing and phylogenetic analysis

All PCR positive samples were subsequently purified and subjected to Sanger sequencing at the Eurofins facilities. The obtained sequences were assessed for quality, aligned in MEGA 12.0.14 software (www.megasoftware.net) using the ClustalW algorithm. All sequences were assembled and compared with similar sequences retrieved from the GenBank nucleotide database using BLASTn. The obtained alignments were used to construct maximum likelihood phylogenetic trees in MEGA using 10,000 bootstrap replicates. Phylogenetic relationships were inferred using the Maximum Likelihood method based on the Tamura-Nei model [24].

### 2.6 Statistical analysis

Given the exploratory nature of this multicenter participatory pilot study and the limited sample size, analyses were primarily descriptive. Categorical variables were summarized as counts and percentages. Exact 95% confidence intervals were calculated where appropriate. No formal statistical inference regarding infestation risk factors or population-level associations was performed.

## 3 Results

### 3.1 Dogs

**Fig. 2:**
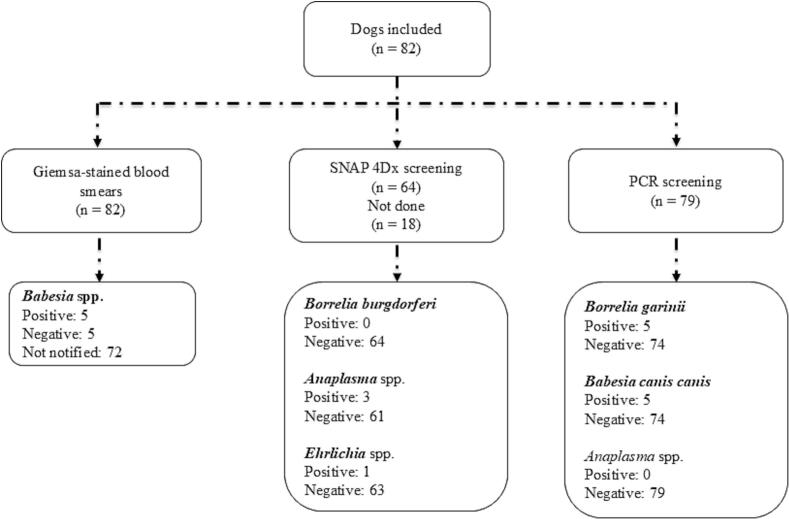
Flow diagram summarizing the results of microscopic, serological, and molecular screening for tick-borne pathogens among the 82 dogs included in the study.

**Fig. 3:**
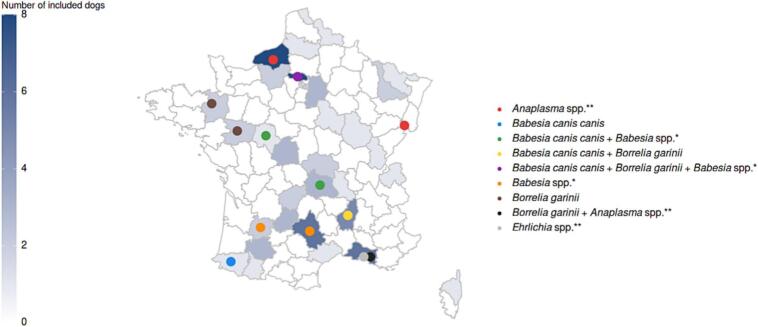
Geographical distribution of tick-borne pathogens (TBPs) detected in dog blood samples using blood smear examination, the SNAP 4Dx Plus test, and/or PCR. The map shows the French administrative departments and the locations where tick-infested dogs were sampled. White areas indicate provinces where no dogs were included in the study. * = positive result using blood smear; ** = positive result using SNAP 4Dx Plus test.

A total of 82 dogs infested with ticks were examined and sampled (Fig. 2). The animals were recruited from 41 veterinary clinics located in 34 departments (Fig. 3). Among them, 28 departments with one clinic each, 5 with two clinics, and one with three clinics (Supplementary Table 2). Among the 75 dogs for which sex information was available, 51 (68%; 95% CI: 57–78) were females and 24 (32%; 95% CI: 23–43) were males. Sex information was missing for seven dogs. Molecular analyses were performed on 79 dogs (Fig. 2), as no blood/serum sample was received for one enrolled dog and empty blood collection tubes were received for two additional dogs (Supplementary Table 2).

Thirteen dogs (13/82; 16%; 95% CI: 9.5–25) tested positive for at least one TBP using molecular, microscopic, and/or serological assays (Table 1). Ten dogs presented a single infection, including five infected with *B. canis canis* and two with *B. garinii* detected by molecular and/or microscopic analyses, as well as two dogs seropositive for *Anaplasma* spp. and one seropositive for *Ehrlichia* spp. Three dogs presented co-infections, including two dogs positive for *Babesia* spp. and *B. garinii* and one dog positive for *B. garinii* and seropositive for *Anaplasma* spp. (Table 2).

**Table 1:** Summary of clinical signs and diagnostic results in dogs (*n* = 13) tested positive for tick-borne pathogens.

Dog ID	Clinical findings	Serological findings	Blood smear	Molecular findings	TBP-positive ticks collected from the dog (engorgement status)
#008	None	Pos for: *Ehrlichia* spp.	Neg	Neg	Neg
#013	None	Pos for: *Anaplasma* spp.	Neg	Pos for: *B. garinii*	Pos for: *R. massiliae* (semi-engorged ticks)
#041	Data missing	Neg	Pos	Pos for: *B. canis canis*	Pos for: *B. canis canis* (engorged tick)
#077	Data missing	Neg	Pos	Negative	Neg
#109	Hemolysis, hematuria	Neg	Pos	Pos for: *B. canis canis*	Neg
#120	Fever	Neg	Pos	Pos for: *B. canis canis* *B. garinii*	Neg
#171	Data missing	Data missing	Not notified	Pos for: *B. garinii*	Neg
#227	Fever, atony (marked lethargy)	Neg	Pos	Negative	Pos for: *B. venatorum* + *B. garinii* (semi-engorged tick)
#261	None	Pos for: *Anaplasma* spp.	Neg	Negative	/
#373	Data missing	Data missing	Not notified	Pos for: *B. canis canis*	Neg
#488	Anemia, fever, hemolysis, icterus, thrombocytopenia	Neg	Neg	Pos for: *B. canis canis* *B. garinii*	Neg
#516	None	Post for: *Anaplasma* spp.	Neg	Neg	Pos for: *B. garinii* (engorged tick)
#523	Data missing	Data missing	Not notified	Pos for: *B. garinii*	Pos for: *B. garinii* (non-engorged ticks)

Neg: negative; Pos: positive.

**Table 2:** : Tick-borne pathogens detected in dogs, infection status (single infection or co-infection), and diagnostic methods used.

Single infection	Diagnostic method	No. dogs
*B. canis canis*	PCR and/or blood smear	5
*B. garinii*	PCR	2
*Anaplasma* spp.	SNAP 4Dx plus	2
*Ehrlichia* spp.	SNAP 4Dx plus	1
Co-infection		
*Babesia* spp. + *B. garinii*	PCR	2
*Anaplasma* spp. + *B. garinii*	SNAP 4Dx plus + PCR	1

Clinical records for five positive dogs were incomplete and were therefore excluded from the analysis of clinical signs. Among the remaining eight dogs, four (#109, #227, #120, and #488) exhibited at least one clinical sign compatible with a tick-borne infection (Table 1), whereas the other four showed no clinical abnormalities despite testing positive for one or more TBPs. The most commonly reported clinical findings were fever (*n* = 3), hematuria and thrombocytopenia (*n* = 2), lethargy (*n* = 1), and anemia (n = 1).

Four dogs (30.8%; 95% CI: 13–58) were seropositive on the SNAP 4Dx test (three for *Anaplasma* spp. and one for *Ehrlichia* spp.), whereas blood smear examination and/or PCR screening detected *Babesia* spp. in seven dogs (53.8% 95% CI: 29–77) (Table 1). All dogs tested negative for *Dirofilaria* spp. antigen using the SNAP 4Dx test.

### 3.2 Ticks

A total of 165 ticks were collected from 80 dogs (tick samples collected from two dogs were not received). Most dogs were infested by a single tick (61.3%), while others carried two (18.8%), three (7.5%), or four ticks (3.8%). High infestation levels were rare, with 5% (4/80) of dogs harboring five to six ticks and 3.8% (3/80) carrying more than eight ticks.

Among the collected ticks, 151 specimens (91.5%; 95% CI: 87–95) were adults, including 108 females and 43 males, while 11 (6.7%; 95% CI: 4–12) were nymphs; no larvae were identified. Overall, three tick genera and five species were identified (Table 3). The most frequently detected species was *D. reticulatus* (29.7%; 49/165; 95% CI: 23–37), followed by *I. ricinus* (18.2%; 30/165; 95% CI: 13–25), *Rh. sanguineus* s.l. (16.4%; 27/165; 95% CI: 11.5–23), *I. hexagonus* (6.7%; 11/165; 95% CI: 4–12), and *D. marginatus* (0.6%; 1/165; 95% CI: 0.1–3). *D. reticulatus* and *I. ricinus* were the most commonly collected tick species during the study period.

**Table 3:** Tick-borne pathogens detected in ticks removed from dogs, by tick species and developmental stage.

Tick species (total)	Developmental stage	PCR-positive /tested	Detected microorganisms (number)
*Dermacentor reticulatus* (*n* = 49)	Adult female	6/ 23	*Babesia canis canis* (n = 3) *Borrelia garinii* (n = 1) *Rickettsia raoultii* (n = 2)
	Adult male	4 / 26	*Babesia canis canis* (n = 1) *Borrelia garinii* (n = 1) *Rickettsia raoultii* (*n* = 2)
*Dermacentor marginatus* (n = 1)	Adult female	0/ 1	–
*Dermacentor* spp. (n = 14)	Adult female	0 / 11	–
	Adult male	0 / 1	–
	Not identified	1 / 2	*Borrelia garinii* (n = 1)
*Ixodes hexagonus* (n = 11)	Nymphs	0 / 11	–
*Ixodes ricinus* (*n* = 30)	Adult female	5 / 26	*Babesia canis canis* (n = 1) *Babesia venatorum* (n = 1) *Borrelia garinii* (n = 2) *Borrelia garinii* + *Babesia venatorum* (n = 1)
	Adult male	0 / 4	–
*Ixodes* spp. (*n* = 12)	Adult female	3 / 12	*Babesia canis canis* (n = 1) *Babesia venatorum* (n = 1) *Borrelia garinii* (n = 1)
*Rhipicephalus sanguineus* s.l. (*n* = 27)	Adult female	5 / 16	*Rickettsia massiliae* (n = 5)
	Adult male	0 / 11	–
*Rhipicephalus* spp. (*n* = 6)	Adult female	2 / 5	*Rickettsia massiliae* (n = 2)
	Adult male	0 / 1	–
Not identified (*n* = 15)	Adult female	1 / 13	*Babesia canis canis* (n = 1)
	Nymph	0 / 1	–
	Not identified	0 / 1	–
Total (*n* = 165)	Adult female	22 / 108 (20.4%)	*Babesia canis canis* (n = 6) *Babesia venatorum* (n = 2) *Borrelia garinii* (n = 4) *Rickettsia massiliae* (n = 7) *Rickettsia raoultii* (n = 2) *Borrelia garinii* + *Babesia venatorum* (n = 1)
	Adult male	4 / 43 (9.3%)	*Babesia canis canis* (n = 1) *Borrelia garinii* (n = 1) *Rickettsia raoultii* (n = 2)
	Nymph	0 / 12	–
	Not identified	1/ 3	*Borrelia garinii* (n = 1)

Mixed infestations were observed in three dogs (3.8%; 3/80; 95% CI: 1.3–10.5), which harbored both *D. reticulatus* and *I. ricinus*. Among the collected ticks, 108 specimens (65.9%; 95% CI: 58–73) were fully or semi-engorged, whereas 56 (34.1%; 95% CI: 27–42) were non-engorged. Notably, five male *D. reticulatus* ticks (19.2%; 95% CI: 8.5–38 of males) were classified as semi-engorged.

**Fig. 4:**
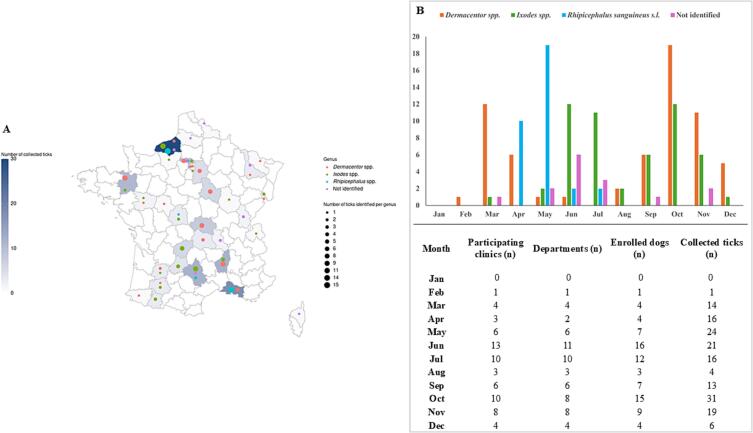
Geographical (A) and seasonal distribution (B) of ticks collected from dogs during the study. Seasonal data are presented as the total number of ticks recorded per month. The table below panel B summarizes the monthly numbers of participating clinics, represented departments, enrolled dogs, and collected ticks.

Regarding the geographic distribution of submissions, tick samples were received from multiple regions across France, although the number of submissions varied among departments (Fig. 4A). *Dermacentor* spp., *Ixodes* spp., and *Rh. sanguineus* s.l. were identified in several regions, and multiple tick genera were detected within the same departments (Fig. 4A).

The number of tick submissions varied throughout the study period and among tick genera (Fig. 4B). *Dermacentor* spp. were most frequently submitted in March and October, *Ixodes* spp. in June and July, and *Rh. sanguineus* s.l. between April and July, with the highest number of submissions recorded in May (Fig. 4B).

### 3.3 Tick-borne pathogens and endosymbionts

Following tick identification, we analyzed the collected specimens for the presence of microorganisms and TBPs. Of 165 specimens analyzed, 98 (59.4%) were positive for TBPs and/or endosymbionts by PCR. Among all specimens, 28 (17%; 95% CI: 12–23) were positive for at least one TBP (Table 3). Among the detected TBPs, *B. canis and B. garinii* were identified in seven and eight ticks, respectively. In addition, three *I. ricinus* specimen was found positive for *B. venatorum. Rickettsia* spp. was identified in 11 specimens, including *Rh. sanguineus* s.l. (*n* = 7) and *Dermacentor* spp. (*n* = 4). The species identified were *R. massiliae* (4.2%; 7/165; 95% CI: 2–8.5) and *R. raoultii* (2.4%; 4/165; 95% CI: 1–6) (Table 3). Co-detection of various endosymbionts and/or environmental microbiota was observed in 5.5% of specimens (9/165; 95% CI: 3–10), as shown in Supplementary Table 3.

All DNA-positive samples were sequenced. The resulting consensus sequences from the forward and reverse reads were deposited in the GenBank database, and the corresponding accession numbers are provided in Supplementary Table 4. These sequences were used to infer a phylogenetic tree using the maximum likelihood method.

**Fig. 5:**
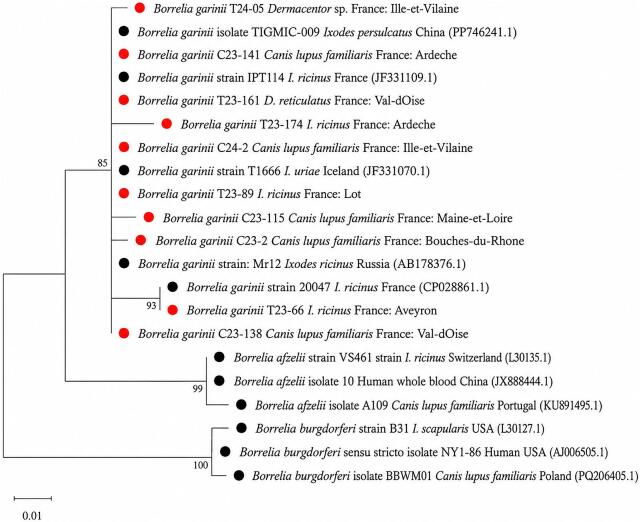
Maximum Likelihood phylogenetic tree inferred from partial 5S-23S rRNA intergenic spacer sequences (∼250 bp) of *Borrelia burgdorferi* sensu lato detected in the present study. The tree depicts the phylogenetic relationships among 21 *B. burgdorferi* s.l. isolates. Each sequence is identified by its strain name, species, host, and country of origin. Sequences obtained in the present study (*n* = 10) are highlighted by red circles, whereas reference sequences retrieved from GenBank (*n* = 11) are indicated by dark circles together with their corresponding accession numbers. Bootstrap values were calculated from 10,000 replicates, and only values >70% are shown. (For interpretation of the references to colour in this figure legend, the reader is referred to the web version of this article.)

Comparative analysis of the twelve *5S-23S rRNA* intergenic spacer sequences (∼250 bp) demonstrated a high level of similarity (99–100%) with *B. garinii* sequences available in the GenBank database (PP746241, AB178376, JF331109) (Fig. 5).

**Fig. 6:**
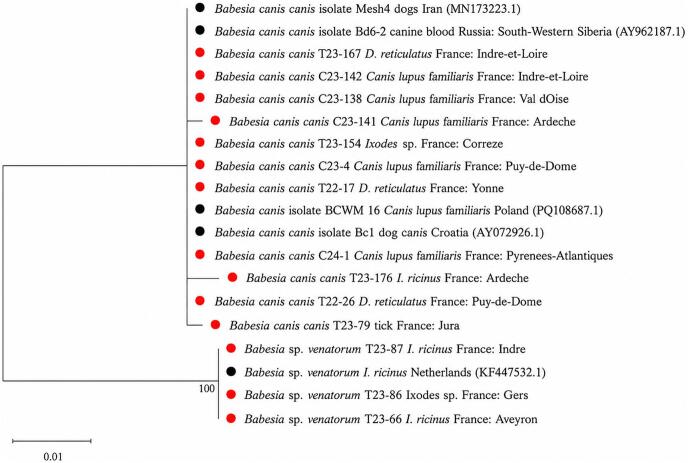
Maximum Likelihood phylogenetic tree inferred from partial 18S rRNA gene sequences (∼550 bp) of *Babesia* spp. detected in the present study. The tree depicts the phylogenetic relationships among 19 *Babesia* species. Each sequence is labeled with the strain designation, species name, host, and country of origin. Sequences obtained in the present study (*n* = 14) are highlighted by red symbols, whereas reference sequences retrieved from GenBank (*n* = 5) are indicated by dark symbols together with their corresponding accession numbers. Bootstrap values were calculated from 10,000 replicates, and only values >70% are shown. (For interpretation of the references to colour in this figure legend, the reader is referred to the web version of this article.)

Among the fourteen partial sequences amplified for the *18S rRNA* gene of *Babesia*, 11 /14 (78.6%) showed 99–100% sequence identity with *B. canis canis* (MN173223, AY072926, AY962187) sequences deposited in GenBank. In contrast, the sequences obtained from three (21.4%) other ticks exhibited 99–100% identity with *B. venatorum* (also known as *Babesia* sp. EU1) (KF447532, KU145466), according to BLAST analysis (Fig. 6).

**Fig. 7:**
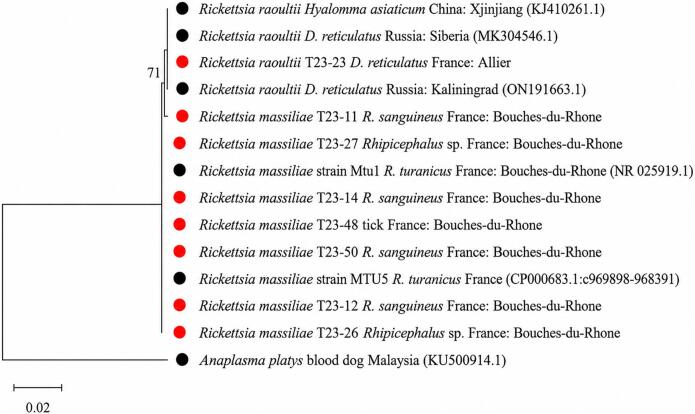
Maximum Likelihood phylogenetic tree inferred from 16S rRNA gene sequences (∼746 bp) of *Rickettsia* spp. detected in the present study (*n* = 8). Sequences of *Rickettsia massiliae* and *Rickettsia raoultii* detected in this study (from ixodid ticks) are highlighted by red circles. These sequences are shown in comparison with reference sequences of other *Rickettsia* species (n = 5) retrieved from GenBank. Reference sequences are indicated by dark circles together with their corresponding accession numbers. *Anaplasma platys* was used as an outgroup for phylogenetic reconstruction. Bootstrap values were calculated from 10,000 replicates, and only values >70% are shown. (For interpretation of the references to colour in this figure legend, the reader is referred to the web version of this article.)

Among the eight sequences amplified for the 16S rRNA gene, one sequence showed 100% identity with *R. raoultii* (KJ410261, MK304546, ON191663), while the remaining seven exhibited 99–100% identity with *R. massiliae* (NR_025919, CP000683) sequences available in GenBank (Fig. 7).

**Fig. 8:**
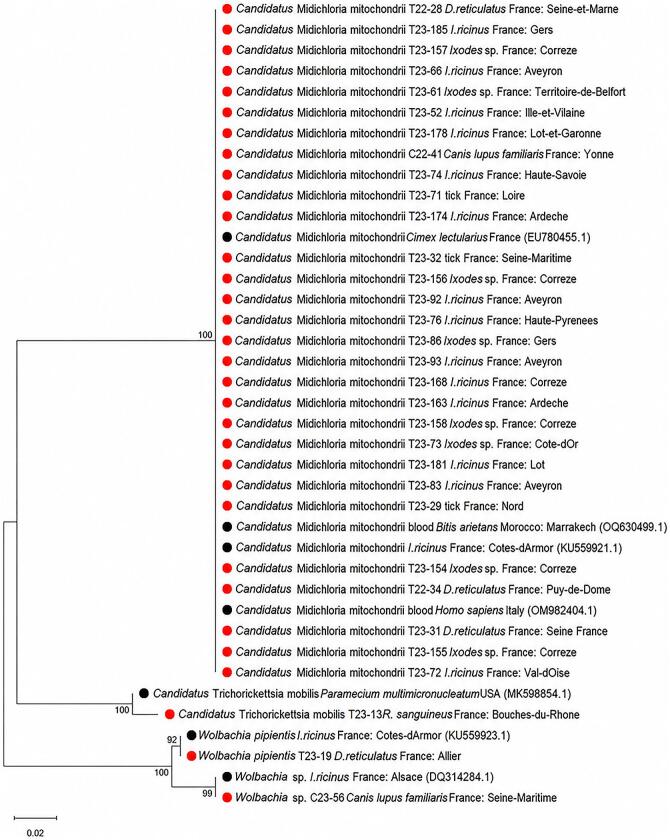
Maximum Likelihood phylogenetic tree inferred from 16S rRNA gene sequences of endosymbionts detected in the present study (*n* = 32). Sequences of *Candidatus Trichorickettsia mobilis*, *Wolbachia pipientis*, *Wolbachia* spp., and *Candidatus Midichloria mitochondrii* detected in this study (from ixodid ticks or dog blood) are highlighted by red circles. These sequences are shown in relation to reference sequences belonging to the order Rickettsiales (*n* = 7), highlighted by dark circles, retrieved from GenBank. Bootstrap values were calculated from 10,000 replicates, and only values >70% are displayed. (For interpretation of the references to colour in this figure legend, the reader is referred to the web version of this article.)

Sequencing analysis of non-pathogenic bacterial amplicons identified several bacterial taxa (Fig. 8). Most sequences (*n* = 29) were obtained from tick samples and showed 100% identity with *Candidatus Midichloria mitochondrii* reference sequences available in GenBank (KU559921, OQ630499, and AY776167). One tick-derived sequence showed 98.8% identity with *Candidatus Trichorickettsia mobilis* (CP112932, HG315610, and MK598854.1), and one tick-derived sequence showed 100% identity with *Wolbachia pipientis* (KU559923). In addition, one *Wolbachia* spp. sequence obtained from a dog blood sample showed 100% identity with the GenBank reference sequence DQ314284 (Fig. 8).

Several non-pathogenic endosymbionts were incidentally detected during the molecular analyses of ticks (Supplementary Table 3). Among the 165 ticks analyzed, bacterial DNA corresponding to endosymbiont-associated taxa and environmental bacteria was identified in 29 samples (17.6%). Detection frequencies varied according to tick species and developmental stage (Supplementary Table 3 and 5). The identified bacteria included members of the genera *Bacillus* (e.g., *B. thuringiensis*, *B. toyonensis*, *B. paranthracis*), *Serratia* (including *Serratia marcescens*), *Conexibacter*, *Phenylobacterium*, *Enterococcus*, *Hydrocarboniphaga*, as well as several uncultured bacterial lineages (Supplementary Table 5). These taxa were detected in multiple tick species, including *D. reticulatus*, *I. ricinus*, and *I. hexagonus*.

## 4 Discussion

To our knowledge, this study is the first in France to implement a multicenter One Health participatory surveillance approach involving multiple stakeholders, including dog owners, the veterinary clinic network Mon Véto, the Institut Pasteur, and the veterinary pharmaceutical company Ceva Santé Animale. Beyond its descriptive findings, this pilot study provides proof-of-concept for the feasibility of veterinary network-based surveillance and highlights its potential value for monitoring pathogen circulation at the human–animal–environment interface.

The predominance of adult ticks observed in this study (91.5%) is consistent with previous reports [25], [26], [27] and may partly reflect a detection bias related to the difficulty of identifying immature stages, which are smaller and often concealed within the fur [28]. This bias may also have been reinforced by the sampling approach, as ticks were collected by veterinarians during clinical examinations rather than through a standardized whole-body tick search, potentially resulting in the underdetection of larvae and nymphs. Although the study was based on a participatory surveillance network, the tick species identified, including *D. reticulatus*, *I. ricinus*, and *R. sanguineus* s.l., are consistent with those commonly reported on dogs in Europe [29], [30].

Temporal variations in the submission of different tick species were observed throughout the study period. While these data do not provide a direct measure of tick activity, the frequent detection of *D. reticulatus* during several months of the year is consistent with previous reports describing extended periods of activity for this species in Europe [31], [32]. These observations further support the importance of maintaining tick prevention and control measures in dogs throughout the year.

The tick burden among dogs was heterogeneous, with most dogs (61.2%; 49/80) carrying a single tick and only a small proportion (5%; 4/80) harboring mixed-species infestations. Similar low frequencies (2–6.7%) of mixed-species infestations in dogs have been reported in previous studies [12], [18], [32], suggesting that the findings observed in our study are broadly consistent with previous observations in Europe.

More than 65% of ticks removed from dogs were either fully or partially engorged, indicating that many ticks had remained attached long enough to initiate blood feeding before removal. Similar observations have been reported in dogs from several European countries [25], [33], [34]. Although the reasons underlying these observations could not be assessed in the present study, the high proportion of engorged ticks highlights the potential for pathogen transmission, as transmission of certain TBPs may occur shortly after tick attachment under field conditions [4]. These observations support the importance of preventive measures aimed at reducing tick attachment and exposure in dogs, including acaricidal treatments, regular tick inspections with prompt removal, environmental management, and owner education. Where available, vaccination against specific tick-borne diseases, such as LB in endemic areas, may provide an additional layer of protection.

The detection of *Rh. sanguineus* s.l. in several participating regions and during multiple months of the study period is noteworthy, as this tick is closely associated with dogs and domestic or peri-domestic environments. The distribution and epidemiological importance of *Rh. sanguineus* s.l. in Europe have been widely discussed in relation to climate change, urbanization, and the movement of dogs [35], [36], [37].

The tick *I. hexagonus* is typically associated with hedgehogs, but it readily parasitizes a variety of mammals, including cats and dogs. Its relatively low frequency in the present study (6.7%) is consistent with previous reports, which indicate that this species typically accounts for only 2–6% of ticks collected from dogs [12], [32], [38], [39]. *I. hexagonus* has been reported to harbor several zoonotic pathogens, including *B. burgdorferi* s.l., *A. phagocytophilum*, and *Rickettsia* spp. [11]. Therefore, although infrequently detected, its potential contribution to pathogen maintenance and transmission within wildlife and domestic animal communities remains relevant and merits further investigation.

No *Hyalomma* ticks were detected in the present study. Nevertheless, their expanding distribution in parts of Europe and their recognized role as vectors of zoonotic pathogens support the need for continued surveillance to better understand their occurrence, host associations, and pathogen carriage.

In our cohort, 15.9% (13/82) of dogs tested positive for at least one TBP using molecular, microscopic, and/or serological methods, including *B. canis canis*, *B. garinii*, *Anaplasma* spp., and *Ehrlichia* spp. Three dogs showed evidence of co-detection involving more than one pathogen. Clinical presentations were variable, and four of the eight dogs for which complete clinical information was available were asymptomatic, highlighting the occurrence of subclinical infections. In Europe, unlike in North America, routine screening of apparently healthy dogs for VBPs is not commonly performed and may warrant further consideration.

The detection of *B. garinii* DNA in five dogs (6.3%; 5/79), despite negative SNAP 4Dx results should be interpreted with caution. As the SNAP 4Dx assay is based on the C6 peptide of *B. burgdorferi* ss, discrepancies between serological and molecular findings may occur when infections involve other *Borrelia* genospecies, such as *B. garinii*. In addition, differences between PCR and serological results may reflect the timing of infection and host immune responses. For example, dogs sampled during the early stages of infection may harbor detectable *Borrelia* DNA before developing a measurable antibody response, whereas seropositive dogs may no longer have detectable circulating bacterial DNA. Because only a single sample was available from each dog, the temporal relationship between pathogen detection and seroconversion could not be assessed.

Importantly, PCR detection of *B. garinii* DNA in blood does not, by itself, establish a diagnosis of clinical LB. Indeed, at least one PCR-positive dog was asymptomatic, and clinical information was incomplete for some animals. Nevertheless, the detection of *B. garinii* in both dogs and attached ticks indicates exposure to this pathogen and is consistent with previous studies showing that *B. garinii* is among the most frequently detected *Borrelia* genospecies in ticks collected from dogs in Europe [40], [41]. Finally, it is noteworthy that *B. garinii* has occasionally been detected in dogs presenting with clinical signs compatible with LB, including fever, anorexia, and locomotor disorders [42], [43].

The molecular detection of *B. canis canis* in five dogs (6.3%; 5/79) confirms the presence of this pathogen among the enrolled dogs and is consistent with its recognized importance as a cause of canine babesiosis in Europe. Molecular epidemiological studies have documented the circulation of several *Babesia* species in French dogs. For example, René-Martellet et al. reported PCR prevalences of 13.6% for *B. vogeli* and 12.9% for *B. canis* in dogs from southern France in 2015 [44]. Clinical presentations among positive dogs were variable, ranging from asymptomatic infection to signs compatible with babesiosis, including fever, anemia, thrombocytopenia, and hematuria. Previous studies have shown that *B. canis* infections may be associated with nonspecific clinical signs such as lethargy, fever, and pale or icteric mucous membranes, while severe cases may develop hemolytic anemia and pigmenturia [45], [46], [47]. Co-detection of *B. canis canis* and *B. garinii* in two dogs further illustrates the complexity of exposure to TBPs in naturally exposed canine populations.

In addition, the absence of *B. canis* DNA in ticks collected from most PCR-positive dogs may reflect several factors, including prior tick detachment, differences in the timing of pathogen acquisition and transmission, or differences in detection sensitivity between host blood samples and tick specimens. Interpretation of host–tick concordance should therefore be made with caution, particularly because most ticks were collected while feeding and the detection of pathogen DNA in ticks does not necessarily indicate established tick infection. Despite the increasing number of autochthonous canine babesiosis cases reported in Europe, detection of *B. canis* in tick populations remains relatively infrequent and highly variable between regions. For example, in southern France, *B. canis* DNA was detected in 9.7% of *D. reticulatus* ticks and 1.6% of *Rh. sanguineus* ticks [44]. Similarly, the prevalence of *B. canis* in *D. reticulatus* ranged from 0% in western regions to 14.7% in eastern Slovakia [48], and from 0% in western areas to 5.9% in eastern Poland [49]. In Germany, studies of *D. reticulatus* collected from vegetation and dogs in endemic foci reported prevalence rates of up to 0.3% and 0.08%, respectively [50], [51].

In the present study, antibodies against *Anaplasma* spp. and *Ehrlichia* spp. were detected in three and one dogs, respectively, using the SNAP 4Dx Plus test, whereas all dogs tested negative by PCR. Most seropositive dogs were asymptomatic, consistent with previous reports describing subclinical or past infections [52] Several studies comparing rapid serological assays, such as the SNAP 4Dx Plus test, with PCR-based methods have reported discrepancies between serological and molecular results, reflecting differences in the stage of infection and the host immune response [53], [54]. Accordingly, the positive SNAP 4Dx results observed in this study likely indicate previous exposure rather than active infection at the time of sampling. Furthermore, no direct relationship could be established between tick infestation at the time of examination and serological findings, as antibody detection reflects past exposure to TBPs rather than recent tick attachment [55].

Several zoonotic and veterinary-relevant TBPs were detected in ticks collected from dogs, including *B. garinii*, *R. massiliae*, *R. raoultii*, and *B. venatorum*. However, these findings should be interpreted with caution. Because most ticks analyzed were collected while feeding on dogs, the detection of pathogen DNA does not necessarily indicate established infection of the tick and may, in some cases, reflect the presence of pathogen DNA in the host blood meal.

The identification of spotted fever group *Rickettsia* in ticks removed from dogs is particularly important because rickettsioses may be underdiagnosed due to nonspecific clinical presentations and variable clinician awareness [56], [57]. *Rickettsia massiliae*, mainly associated with *Rhipicephalus* ticks, is a recognized human pathogen reported in Mediterranean and broader European settings, including France, and can cause Mediterranean spotted fever like disease [57], [58]. Likewise, the detection of *R. raoultii* (commonly linked to *Dermacentor* spp.) is consistent with the growing recognition of *Dermacentor*-associated rickettsioses in Europe, which can present with characteristic eschars and lymphadenopathy syndromes [57]. Although our study was not designed to validate dogs as sentinels through comparison with human surveillance data, these findings support the potential value of dogs and veterinary networks as complementary sources of information on the circulation of TBPs. In addition, because of their close contact with humans, dogs may contribute to human exposure by introducing ticks into domestic environments, as previously highlighted in European studies on companion animals [59], [60].

Although no dogs tested positive in this study, dogs are known to be susceptible to tick-borne rickettsial infections, particularly those caused by *R. conorii*, with reported seroprevalence varying considerably according to region and local tick activity. For example, studies conducted in Portugal have reported seroprevalence rates of approximately 27% in dogs [61]. Even when infections remain subclinical, dogs may be frequently exposed to infected ticks. These observations support the potential value of veterinary surveillance networks for improving our understanding of TBP circulation in endemic areas.

The detection of *B. garinii* is noteworthy, as this species is a well-established cause of human LB in Europe and is frequently associated with neurological manifestations [62]. Its circulation is closely linked to the distribution of *I. ricinus* [63], [64]. Although dogs are not considered reservoir hosts for *Borrelia* spirochetes, the detection of *B. garinii* DNA in dog blood and in ticks collected from dogs indicates exposure to this pathogen and the presence of competent vectors in the study area. Similarly, the identification of *B. venatorum* (formerly *Babesia* sp. EU1) in *Ixodes* ticks deserves attention because this species can cause human babesiosis, particularly in immunocompromised individuals, and has been reported throughout Europe [65]. Although no conclusions regarding human transmission can be drawn from the present study, the detection of zoonotic *Borrelia*, *Rickettsia*, and *Babesia* species in dog-associated ticks supports the potential value of integrated One Health surveillance approaches for improving our understanding of pathogen circulation in shared environments.

Several endosymbionts and environmental bacteria were incidentally detected during the molecular analyses. Because the study was not designed to characterize tick microbiota, these findings should be interpreted with caution. Among the detected taxa, *Candidatus* Midichloria mitochondrii, *Francisella*-like endosymbionts, *Coxiella*-like endosymbionts, *Wolbachia*, and *Candidatus* Trichorickettsia mobilis have previously been reported in ticks and other arthropods [66], [67].

Several limitations should be considered when interpreting the findings of this study. First, the sample size was relatively small, and the geographic distribution of submissions was uneven, reflecting differences in participation among veterinary clinics rather than a standardized sampling framework. Furthermore, only tick-infested dogs presented to participating veterinary clinics were enrolled, which may have introduced selection bias and limits the representativeness of the study population. The exploratory cross-sectional design and the absence of longitudinal follow-up precluded the assessment of infection dynamics, pathogen persistence, and clinical outcomes over time. Additional methodological limitations should also be acknowledged. Nested PCR was selected because of its high analytical sensitivity for detecting low levels of pathogen DNA. However, the use of conventional and nested PCR assays, rather than quantitative PCR, did not allow the estimation of pathogen load or the differentiation between low- and high-intensity infections.

Importantly, this study provides proof-of-concept for a multicenter participatory surveillance approach based on veterinary networks. The involvement of 41 veterinary clinics across 34 French departments, combined with standardized sampling procedures and centralized laboratory analyses, illustrates the practical feasibility of implementing a coordinated One Health surveillance system. Consequently, the findings support the potential value of larger multicenter surveillance programs for monitoring tick species and TBP circulation.

Dogs represent a potentially valuable source of information on the circulation of TBPs because of their frequent exposure to ticks and their close association with human environments. However, our study was not designed to validate dogs as sentinels through comparison with human surveillance data. Future studies integrating veterinary, entomological, and human health data would be required to assess the contribution of dogs to integrated surveillance systems.

In conclusion, this multicenter participatory pilot study supports the feasibility and potential value of integrated surveillance strategies for TBPs in France. By leveraging veterinary networks within a One Health framework, such approaches may contribute to a better understanding of pathogen circulation and help inform future surveillance initiatives.

## Declaration of competing of interest

Several authors are employees of Ceva Santé Animale, and one author is an employee of the Mon Véto veterinary network. These organizations provided logistical support to the study, including the distribution of sample collection kits and coordination of participating veterinary clinics, as described in the manuscript.

## CRediT authorship contribution statement

**Djamel Tahir:** Writing – review & editing, Writing – original draft, Methodology, Formal analysis, Data curation, Conceptualization. **Sophie Dupuis:** Methodology, Formal analysis. **Virginie Geolier-Lapeyronie:** Methodology, Formal analysis, Data curation. **Ambre Sibari:** Writing – review & editing, Formal analysis, Data curation. **Yousra El-Ouatik:** Formal analysis, Data curation. **Cécile Collignon:** Data curation. **Samantha Favy:** Data curation. **Thomas Blondel:** Methodology, Formal analysis. **Alessia Crippa:** Data curation. **Laurence Malandrin:** Writing – review & editing, Methodology. **Elisabeth Ferquel:** Writing – review & editing, Writing – original draft, Validation, Methodology, Formal analysis, Data curation, Conceptualization. **Marie Varloud:** Writing – review & editing, Validation, Project administration, Methodology, Formal analysis, Data curation, Conceptualization. **Valérie Choumet:** Writing – review & editing, Writing – original draft, Validation, Project administration, Methodology, Formal analysis, Data curation, Conceptualization.

## Ethics approval

All experimental protocols in this study were ethically approved by the Avogadro LS Ethics Committee (062CE-CEEA, France) under notification number 2022–00928.

## Author statement

All authors have accepted responsibility for the entire content of this manuscript and consented its submission to the journal, reviewed the results, and approved the final version of the manuscript. We declare that this manuscript is original, has not been published before, and is not currently being considered for publication elsewhere.

## Funding

This research did not receive any specific grant from funding agencies in the public, commercial, or not-for-profit sectors.

## Declaration of competing interest

The authors declare that they have no known competing financial interests or personal relationships that could have appeared to influence the work reported in this paper.

## Data Availability

All data generated during this study are included in the main manuscript and the supplementary material.
