# Supplementary material for: Tick-borne pathogens in dogs and their ticks in France: Molecular and serological evidence from a multicenter participatory study
Source: One Health. 2026 Jun 17;23:101487. doi: 10.1016/j.onehlt.2026.101487 (PMC13324308; doi:10.1016/j.onehlt.2026.101487)
Supplement: Supplementary file 1 — Supplementary material [file mmc1.docx]

**Supplementary materials**

**Supplementary Table 1**. Primers used in this study and PCR conditions (annealing temperature).

| Microorganisms | Gene | Name | Sequences 5′-3′ | Annealing temperature (°C) | base pairs | References |
| --- | --- | --- | --- | --- | --- | --- |
| *Borrelia burgdorferi* s.l. | 5S-23S ribosomal RNA (rRNA) | INS1  S23R  RRC  RRB | GAAAAGAGGAAACACCTGTT  TCGGTAATCTTGGGATCAAT  CTGCGAGTTCGCGGGAGAG  AAGCTCCTAGGCATTCACCATA | 56  59 | 257 | [68] |
| *Anaplasma* spp.  *Ehrlichia* spp.  *Rickettsia* spp.  Microbiomes | universal 16S rRNA | EHR521  EHR747 | TGTAGGCGGTTCGGTAAGTTAAAG  GCACTCATCGTTTACAGCGTG | 55 | 247 | [69] |
|  |  | EHR16SD  EHR16SR | GGTACCYACAGAAGAAGTCC  TAGCACTCATCGTTTACAGC | 55 | 345 | [70] |
|  |  | Platys-F  Platys-R | GATTTTTGTCGTAGCTTGCTATG  TAGCACTCATCGTTTACAGC | 56 | 380 | [71] |
| *Babesia* spp. | 18S rRNA | BabGR2  BabGF2ter | CCAAAGACTTTGATTTCTCTC  TTGTAATTGGAATGATGGTGA | 57 | 559 | [72] |

**References**

[68] N. Sertour, V. Cotté, M. Garnier, L. Malandrin, E. Ferquel, V. Choumet, Infection Kinetics and Tropism of *Borrelia burgdorferi* sensu lato in Mouse After Natural (via Ticks) or Artificial (Needle) Infection Depends on the Bacterial Strain., Front. Microbiol. 9 (2018) 1722. https://doi.org/10.3389/fmicb.2018.01722.

[69] S.M. Chen, J.S. Dumler, J.S. Bakken, D.H. Walker, Identification of a granulocytotropic *Ehrlichia* species as the etiologic agent of human disease, J. Clin. Microbiol. 32 (1994) 589–595. https://doi.org/10.1128/jcm.32.3.589-595.1994.

[70] P. Parola, V. Roux, J.-L. Camicas, I. Baradji, P. Brouqui, D. Raoult, Detection of ehrlichiae in African ticks by polymerase chain reaction, Trans. R. Soc. Trop. Med. Hyg. 94 (2000) 707–708. https://doi.org/10.1016/S0035-9203(00)90243-8.

[71] Y. Motoi, H. Satoh, H. Inokuma, T. Kiyuuna, Y. Muramatsu, H. Ueno, Ç. Morita, First Detection of *Ehrlichia platys* in Dogs and Ticks in Okinawa, Japan, Microbiol. Immunol. 45 (2001) 89–91. <https://doi.org/10.1111/j.1348-0421.2001.tb01263.x>.

[72] M. Jouglin, I.G. Fernández-de-Mera, N. de la Cotte, F. Ruiz-Fons, C. Gortázar, E. Moreau, S. Bastian, J. de la Fuente, L. Malandrin, Isolation and characterization of *Babesia pecorum* sp. nov. from farmed red deer (*Cervus elaphus*), Vet. Res. 45 (2014) 78. https://doi.org/10.1186/s13567-014-0078-7.

| **Dog ID**  **Supplementary Table 2**. Tick-borne pathogens and endosymbionts detected in dogs and their ticks, with associated clinical signs, tick species, location, and sex of each dog. | **Sex** | **Dpt. No.** | **Department name** | **Date of reception** | **Clinical Signs** | **Sample tested for PCR** | **SNAP 4Dx Plus result** | **Blood smear result** | **Ectoparasiticide treatment** | **Tick-borne infection in dog** | **Number and species of ticks collected** | **Pathogens/endosymbionts detected in ticks (engorgement status)** |
| --- | --- | --- | --- | --- | --- | --- | --- | --- | --- | --- | --- | --- |
| **#007** | M | 13 | Bouches-du-Rhône | 03/04/2023 | No | Serum | Neg | Not notified | No | Neg | *Rh. sanguineus* s.l. (n = 2) | 1 negative (eng), 1 negative (non-eng) |
| **#008** | M | 13 | Bouches-du-Rhône | 12/09/2023 | No | Serum | Pos for *Ehrlichia* | Neg | Yes | *Ehrlichia* spp. | *D. reticulatus* (n = 3) | 2 negative (eng), 1 negative (non-eng) |
| **#010** | F | 27 | Eure | 09/05/2023 | No | Serum | Neg | Not notified | No | Neg | *I. hexagonus*  (n = 1) | 1 negative (semi-eng) |
| **#012** | F | 13 | Bouches-du-Rhône | 06/12/2023 | No | Serum | Neg | Not notified | Not notified | Neg | *D. reticulatus* (n =1) | 1 *Candidatus* Tisiphia endosymbiont (eng) |
| **#013** | M | 13 | Bouches-du-Rhône | 03/04/2023 | No | Serum | Pos for *Anaplasma* | Neg | No | *Borrelia garinii* + *Anaplasma* spp. | *Rh. sanguineus* s.l.  (n = 5) | 3 *Rickettsia massiliae* (semi-eng), 1 *Candidatus Trichorickettsia mobilis* (semi-eng), 1 negative (non-eng) |
| **#014** | / | 95 | Val-d'Oise | 26/08/2023 | No | Serum | Not notified | Not notified | Not notified | Neg | *D. reticulatus* (n =2) | 2 negative (non-eng) |
| **#015** | M | 95 | Val-d'Oise | 15/11/2023 | No | Serum | Neg | Not notified | No | Neg | not identified (n = 2) | 1 *Francisella-*like endosymbiont (eng), 1 negative (eng) |
| **#025** | F | 35 | Ille-et-Vilaine | 19/05/2023 | Not notified | Blood + serum | Not notified | Not notified | No | Neg | *I. ricinus* (n = 1) | 1 *Candidatus* Midichloria mitochondrii (semi-eng) |
| **#041** | F | 37 | Indre-et-Loire | 18/10/2023 | Not notified | Serum | Neg | Yes piroplasmose | No | *Babesia canis canis* | *D. reticulatus* (n = 1) | 1 *Babesia canis canis* (eng) |
| **#042** | F | 77 | Seine-et-Marne | 18/10/2023 | No | Serum | Neg | Not notified | Yes | Neg | *D. reticulatus* (n = 2) | 1 *Candidatus* Midichloria mitochondrii (non-eng), 1 negative (non-eng) |
| **#043** | F | 77 | Seine-et-Marne | 25/10/2023 | No | Serum | Neg | Not notified | Yes | Neg | *D. reticulatus* (n = 1) | 1 negative (non-eng) |
| **#047** | F | 92 | Hauts-de-Seine | 20/07/2023 | Not notified | Serum | Not notified | Not notified | Not notified | Neg | not identified (n = 1) | 1 negative (eng) |
| **#053** | F | 57 | Moselle | 24/10/2023 | Not notified | Serum | Neg | Not notified | Not notified | Neg | *D. reticulatus* (n =1) | 1 *Francisella*-like endosymbiont (non-eng) |
| **#063** | F | 77 | Seine-et-Marne | 29/03/2023 | No | Serum | Neg | Not notified | Yes | Neg | *D. reticulatus* (n = 2) | 2 negative (non-eng) |
| **#077** | F | 47 | Lot-et-Garonne | 07/11/2023 | Not notified | Serum | Neg | Yes piroplasmose | No | Piroplasmosis (*Babesia* spp.) | *D. reticulatus* (n = 1), *I. ricinus* (n = 1) | 1 *Candidatus* Midichloria mitochondrii (eng), 1 negative (non-eng) |
| **#083** | M | 47 | Lot-et-Garonne | 11/12/2023 | No | Serum | Neg | Not notified | No | Neg | *Dermacentor* spp. (n = 1) | 1 negative (eng) |
| **#088** | M | 46 | Lot | 09/11/2023 | Not notified | Serum | Not notified | Not notified | Not notified | Neg | *I.ricinus* (n = 2) | 1 *Candidatus* Midichloria mitochondrii (eng), 1 negative (non-eng) |
| **#109** | F | 63 | Puy-de-Dôme | 21/02/2023 | Yes | Serum | Neg | Yes piroplasmose | No | *Babesia canis canis* | *Dermacentor* spp. (n = 1) | 1 negative (non-eng) |
| **#117** | M | 94 | Val-de-Marne | 23/11/2023 | No | Serum | Neg | Not notified | Yes | Neg | *D. marginatus* (n = 1) | 1 *Francisella-*like endosymbiont (semi-eng) |
| **#118** | F | 95 | Val-d'Oise | 04/10/2023 | No | Serum | Neg | Not notified | No | Neg | *D. reticulatus* (n =1) | 1 *Borrelia garinii* (eng) |
| **#120** | F | 95 | Val-d'Oise | 29/09/2023 | Yes | Serum | Neg | Yes piroplasmose | No | *Babesia canis canis* + *Borrelia garinii* | *D. reticulatus* (n =1) | 1 negative (eng) |
| **#121** | F | 46 | Lot | 24/07/2023 | No | Serum | Neg | Not notified | No | Neg | *I.ricinus* (n = 1) | 1 *Borrelia garinii* + *Candidatus* Midichloria mitochondrii (semi-eng) |
| **#136** | F | 95 | Val-d'Oise | 24/10/2023 | Not notified | Blood + serum | Not notified | Not notified | Not notified | Neg | *D. reticulatus* (n =1) | 1 negative (eng) |
| **#148** | F | 95 | Val-d'Oise | 30/06/2023 | Not notified | Serum | Not notified | Not notified | Not notified | Neg | *Ixodes* spp. (n = 1) | 1 *Candidatus Midichloria mitochondrii* (semi-eng) |
| **#151** | F | 39 | Jura | 10/07/2023 | No | Blood + serum | Neg | Not notified | Not notified | Neg | not identified (n = 2) | 1 *Babesia canis canis* + *Candidatus* Midichloria mitochondrii (semi-eng), 1 negative (semi-eng) |
| **#157** | F | 20 | Corse | 19/06/2023 | No | Serum | Neg | Not notified | Yes | Neg | not identified (n = 1) | 1 negative (semi-eng) |
| **#166** | F | 49 | Maine-et-Loire | 15/06/2023 | No | Serum | Neg | Not notified | No | Neg | *Dermacentor* spp. (n = 1) | 1 negative (semi-eng) |
| **#171** | F | 49 | Maine-et-Loire | 17/07/2023 | Not notified | Serum | Not notified | Not notified | Not notified | *Borrelia garinii* | *I. ricinus* (n = 1) | 1 negative (semi-eng) |
| **#175** | F | 46 | Lot | 20/07/2023 | No | Serum | Neg | Not notified | Not notified | Neg | *I. ricinus* (n = 1) | 1 *Candidatus* Midichloria mitochondrii (semi-eng) |
| **#185** | M | 94 | Val-de-Marne | 27/12/2023 | No | Serum | Neg | Not notified | Not notified | Neg | *I. hexagonus*  (n = 1) | 1 negative (semi-eng) |
| **#187** | F | 12 | Aveyron | 17/07/2023 | Yes | Serum | Neg | Not notified | No | Neg | *I. ricinus* (n = 1) | 1 *Candidatus* Midichloria mitochondrii (semi-eng) |
| **#188** | M | 12 | Aveyron | 17/07/2023 | No | Serum | Neg | Not notified | Not notified | Neg | *Rh.* *sanguineus* s.l. (n = 2) | 2 negative (semi-eng) |
| **#199** | F | 3 | Allier | 20/04/2023 | No | Serum | Neg | Not notified | Not notified | Neg | *D. reticulatus*  (n = 6) | 1 *Babesia canis canis* (non-eng), 3 *Rickettsia conorii* subsp. *raoultii* (non-eng), 1 negative (eng), 1 negative (non-eng) |
| **#207** | M | 3 | Allier | 08/11/2023 | No | No blood/serum | Neg | Not notified | Not notified | no blood | *D. reticulatus (n = 1)*, *Dermacentor* spp. (n = 1) | 1 negative (eng), 1 negative (non-eng) |
| **#214** | M | 19 | Corrèze | 25/09/2023 | Not notified | Blood | Not notified | Not notified | Not notified | Neg | *Ixodes* spp. (n = 5) | 1 *Babesia canis canis* + *Candidatus* Midichloria mitochondrii (eng) 3 *Candidatus* Midichloria mitochondrii (eng), 1 *Borrelia garinii* + *Candidatus* Midichloria mitochondrii (eng) |
| **#222** | M | 76 | Seine-Maritime | 24/07/2023 | No | Serum | Neg | Not notified | Not notified | Neg | *Ixodes* spp. (n = 1) | 1 *Candidatus* Midichloria mitochondrii (semi-eng) |
| **#227** | F | 12 | Aveyron | 26/06/2023 | Yes | Serum | Neg | Yes piroplasmose | No | Piroplasmosis (*Babesia* spp.) | *I. ricinus* (n = 3) | 1 *Babesia venatorum* + *Borrelia garinii* + *Candidatus* Midichloria mitochondrii (semi-eng) 2 negative (non-eng) |
| **#229** | M | 36 | Indre | 26/06/2023 | Not notified | Serum | Neg | Not notified | Not notified | Neg | *Ixodes* spp. (n = 1) | 1 negative (semi-eng) |
| **#232** | M | 36 | Indre | 19/07/2023 | No | Serum | Neg | Not notified | Not notified | Neg | *I. ricinus*  (n = 1) | 1 *Babesia venatorum* (semi-eng) |
| **#234** | M | 36 | Indre | 23/06/2023 | No | Serum | Neg | Not notified | Not notified | Neg | *R.* *sanguineus* s.l.  (n = 1) | 1 negative (semi-eng) |
| **#249** | M | 76 | Seine-Maritime | 01/08/2023 | Yes | Serum | Neg | Not notified | Yes | Neg | *I. ricinus*  (n = 1) | 1 *Candidatus* Midichloria mitochondrii (eng) |
| **#251** | F | 19 | Corrèze | 20/10/2023 | Not notified | Blood | Neg | Not notified | Not notified | Neg | *I. ricinus*  (n = 1) | 1 *Candidatus* Midichloria mitochondrii (eng) |
| **#257** | F | 12 | Aveyron | 01/08/2023 | No | Serum | Neg | Not notified | Yes | Neg | *I. ricinus*  (n = 1) | 1 negative (eng) |
| **#261** | F | 76 | Seine-Maritime | 13/06/2023 | No | Serum | Pos for *Anaplasma* | Neg | *Yes* | *Anaplasma* spp. | No ticks received | No ticks received |
| **#264** | F | 21 | Côte-d'Or | 30/06/2023 | No | Serum | Neg | Not notified | No | Neg | *Ixodes* spp. (n = 1) | 1 *Candidatus* Midichloria mitochondrii (semi-eng) |
| **#314** | F | 76 | Seine-Maritime | 28/10/2022 | No | Blood + serum | Neg | Not notified | No | Neg | *I. hexagonus*  (n = 9) | 1 *Coxiella* endosymbiont (eng), 8 negative (eng) |
| **#333** | / | 76 | Seine-Maritime | 27/03/2023 | Not notified | Serum | Not notified | Not notified | Not notified | Neg | Not identified (n = 1) | 1 negative |
| **#334** | F | 76 | Seine-Maritime | 07/10/2023 | No | Serum | Neg | Not notified | No | Neg | *D. reticulatus* (n = 2) | 1 *Rickettsia conorii* subsp. *raoultii* (semi-eng), 1 negative (semi-eng) |
| **#340** | F | 76 | Seine-Maritime | 15/05/2023 | No | No blood/serum | Neg | Not notified | Yes | no blood | not identified (n = 1) | 1 *Candidatus* Midichloria mitochondrii (semi-eng) |
| **#371** | F | 74 | Haute-Savoie | 03/07/2023 | Not notified | Serum | Not notified | Not notified | Not notified | Neg | *I. ricinus*  (n = 1) | 1 *Candidatus* Midichloria mitochondrii (semi-eng) |
| **#373** | F | 64 | Pyrénées-Atlantiques | 29/03/2023 | Not notified | Serum | Not notified | Not notified | Not notified | *Babesia canis canis* | *Dermacentor* spp.  (n = 1) | 1 negative (eng) |
| **#380** | F | 13 | Bouches-du-Rhône | 17/05/2023 | Not notified | Serum | Neg | Not notified | Not notified | Neg | *Rh.* *sanguineus* s.l.  (n = 4) | 1 *Rickettsia massiliae* (eng), 1 *Rickettsia massiliae* (non-eng), 1 *Coxiella* endosymbiont (non-eng), 1 negative (non-eng) |
| **#381** | / | 13 | Bouches-du-Rhône | 14/04/2023 | No | Serum | Neg | Not notified | Not notified | Neg | *Rh.* *sanguineus* s.l.  (n = 3) | 2 *Rickettsia massiliae* (semi-eng), 1 negative (non-eng) |
| **#399** | / | 54 | Meurthe-et-Moselle | 29/06/2023 | Not notified | Serum | Not notified | Not notified | Not notified | Neg | not identified (n = 2) | 2 negative (eng) |
| **#413** | / | 54 | Meurthe-et-Moselle | 29/09/2023 | Not notified | Serum | Not notified | Not notified | Not notified | Neg | *D. reticulatus* (n = 1) | 1 *Borrelia garinii* (non-eng) |
| **#414** | M | 95 | Val-d'Oise | 22/06/2023 | Not notified | Serum | Not notified | Not notified | Not notified | Neg | *Rh.* *sanguineus* s.l.  (n = 1) | 1 *Candidatus* Midichloria mitochondrii *(*semi-eng) |
| **#416** | M | 34 | Hérault | 01/06/2023 | Not notified | Serum | Not notified | Not notified | Not notified | Neg | No ticks received | No ticks received |
| **#420** | M | 90 | Territoire de Belfort | 09/06/2023 | No | Serum | Neg | Not notified | Yes | Neg | *Ixodes* spp (n = 1) | 1 *Candidatus* Midichloria mitochondrii (semi-eng) |
| **#428** | F | 59 | Nord | 02/05/2023 | No | Serum | Neg | Not notified | No | Neg | not identified (n = 1) | 1 *Candidatus* Midichloria mitochondrii (semi-eng) |
| **#437** | F | 12 | Aveyron | 13/06/2023 | No | Serum | Neg | Not notified | Not notified | Neg | not identified (n = 1) | 1 negative (eng) |
| **#440** | F | 12 | Aveyron | 12/06/2023 | No | No blood/serum | Neg | Not notified | Not notified | No blood | *I. ricinus* (n = 4) | 2 *Candidatus* Midichloria mitochondrii (semi-eng), 1 negative (semi-eng), 1 negative (non-eng) |
| **#444** | F | 65 | Hautes-Pyrénées | 04/07/2023 | No | Serum | Neg | Not notified | No | Neg | *I. ricinus*  (n = 3) | 2 *Candidatus* Midichloria mitochondrii (semi-eng), 1 negative (non-eng) |
| **#453** | F | 90 | Territoire de Belfort | 04/09/2023 | No | Serum | Neg | Not notified | Yes | Neg | *D. reticulatus* (n = 1) | 1 negative (non-eng) |
| **#456** | F | 76 | Seine-Maritime | 15/05/2023 | No | Serum | Neg | Not notified | Yes | *Wolbachia* spp. | *Rh.* *sanguineus* s.l.  (n = 15) | 6 negative (semi-eng), 9 negative (non-eng) |
| **#459** | F | 80 | Somme | 18/09/2023 | No | Serum | Neg | Not notified | Not notified | Neg | Not identified (n = 1) | 1 negative (eng) |
| **#479** | F | 7 | Ardèche | 25/10/2023 | No | Serum | Neg | Not notified | Not notified | Neg | *I. ricinus*  (n = 1) | 1 *Borrelai garinii* + *Candidatus* Midichloria mitochondrii (eng) |
| **#480** | F | 7 | Ardèche | 25/10/2023 | Yes | Serum | Neg | Not notified | Not notified | Neg | *D. reticulatus* (n = 4) | 4 negative (semi-eng) |
| **#485** | F | 7 | Ardèche | 12/10/2023 | No | Serum | Neg | Not notified | No | Neg | *D. reticulatus*  (n = 1*)*  *I. ricinus* (n = 1) | 1 negative (non-eng), 1 *Candidatus* Midichloria mitochondrii (eng) |
| **#487** | M | 7 | Ardèche | 07/11/2023 | Yes | Serum | Neg | Not notified | Not notified | Neg | *I. ricinus* (n = 2) | 1 *Candidatus* Midichloria mitochondrii (eng), 1 *Babesia canis canis* + *Candidatus* Midichloria mitochondrii (eng) |
| **#488** | M | 7 | Ardèche | 13/10/2023 | Yes | Serum | Neg | Neg | Yes | *Babesia canis canis* + *Borrelia garinii* | *D. reticulatus* (n = 3) | 1 *Francisella*-like endosymbiont (non-eng), 1 *Candidatus* Midichloria mitochondrii (non-eng), 1 négative (non-eng) |
| **#501** | F | 42 | Loire | 22/06/2023 | No | Serum | Invalid test result | Not notified | No | Neg | Not identified (n = 2) | 1 *Candidatus* Midichloria mitochondrii (semi-eng), 1 negative (semi-eng) |
| **#516** | F | 90 | Territoire de Belfort | 18/09/2023 | No | Serum | Pos for *Anaplasma* | Neg | No | *Anaplasma* spp. | *I. ricinus*  (n = 1) | 1 *Borrelia garinii* (eng) |
| **#523** | F | 35 | Ille-et-Vilaine | 29/03/2023 | Not notified | Serum | Not notified | Not notified | Not notified | *Borrelia garinii* | *Dermacentor* spp.  (n = 9) *I. ricinus* (n = 1) | 1 *Borrelia garinii* (non-eng), 1 negative (semi-eng), 3 negative (eng), 5 negative (eng) |
| **#529** | F | 95 | Val-d'Oise | 26/06/2023 | No | Serum | Neg | Not notified | Not notified | Neg | *Ixodes* spp. (n = 1) | 1 *Candidatus* Midichloria mitochondrii (semi-eng) |
| **#555** | / | not specified | not specified | /12/2023 | Not notified | Serum | Not notified | Not notified | Not notified | Neg | *D. reticulatus*  (n = 3) | 1 *Candidatus* Midichloria mitochondrii (semi-eng), 1 negative (semi-eng), 1 negative (non-eng) |
| **#558** | M | 89 | Yonne | 07/11/2023 | No | Serum | Neg | Not notified | No | *Candidatus* Midichloria mitochondrii | *D. reticulatus*  (n = 6) | 1 *Babesia canis canis* (eng), 2 negative (eng), 3 negative (non-eng) |
| **#561** | M | 63 | Puy-de-Dôme | 10/10/2023 | No | Serum | Neg | Not notified | Yes | Neg | *D. reticulatus* (n =1) | 1 *Babesia canis canis* (non-eng) |
| **#562** | F | 63 | Puy-de-Dôme | 10/10/2023 | No | Serum | Neg | Not notified | Yes | Neg | *D. reticulatus* (n =1) | 1 *Candidatus* Midichloria mitochondrii (semi-eng) |
| **#568** | / | 75 | Paris | 10/05/2023 | Not notified | Serum | Not notified | Not notified | Not notified | Neg | *D. reticulatus* (n =1) | 1 *Candidatus* Midichloria mitochondrii (semi-eng) |
| **#569** | F | 32 | Gers | 30/11/2023 | No | Serum | Neg | Not notified | Not notified | Neg | *I. ricinus*  (n = 1) | 1 *Candidatus* Midichloria mitochondrii (non-eng) |
| **#592** | F | 32 | Gers | 17/07/2023 | No | Serum | Neg | Not notified | Not notified | Neg | *Ixodes* spp. (n = 1) | 1 *Babesia venatorum*  + *Candidatus* Midichloria mitochondrii (semi-eng) |
| **#555 bis** | M | 32 | Gers | 30/11/2023 | No | Serum | Neg | Not notified | No | Neg | *D. reticulatus* (n = 1) | 1 *Francisella*-like endosymbiont (semi-eng) |

**Dpt**: Department; **M**: male; **F**: female; **eng**: engorged; **semi-eng**: semi-engorged; **non-eng**: non-engorged; **Pos:** positive; **Neg**: negative.

## Supplementary Table 3. Endosymbionts detected in ticks removed from dogs, by tick species and developmental stage.

| Tick species (total) | Developmental stage | PCR-positive /tested | Detected microorganisms (number) |
| --- | --- | --- | --- |
| *Dermacentor reticulatus*  (n = 49) | Adult female | 7 / 23 | *Candidatus* Midichloria mitochondrii  (n = 4)  *Francisella*-like endosymbionts (n = 2)  *Wolbachia pipientis* (n = 1) |
|  | Adult male | 3 / 26 | *Candidatus* Midichloria mitochondrii  (n = 2)  *Francisella*-like endosymbionts (n = 1) |
| *Dermacentor marginatus*  (n = 1) | Adult female | 1 / 1 | *Francisella*-like endosymbiont (n = 1) |
| *Dermacentor* spp.  (n = 14) | Adult female | 0 / 11 | – |
|  | Adult male | 0 / 1 | – |
|  | Not identified | 0 / 2 | _ |
| *Ixodes hexagonus*  (n = 11) | Nymph | 1 / 10 | *Coxiella* endosymbiont (n = 1) |
| *Ixodes ricinus*  (n = 30) | Adult female | 19 / 26 | *Candidatus* Midichloria mitochondrii  (n = 19) |
|  | Adult male | 0 / 4 | – |
| *Ixodes* spp.  (n = 12) | Adult female | 11 / 12 | *Candidatus* Midichloria mitochondrii  (n = 11) |
| *Rhipicephalus sanguineus* s.l.  (n = 27) | Adult female | 3 / 16 | *Candidatus* Trichorickettsia mobilis  (n = 1)  *Coxiella* endosymbiont (n = 1)  *Candidatus* Midichloria mitochondrii  (n = 1) |
|  | Adult male | 0 / 11 | – |
| *Rhipicephalus* spp.  (n = 6) | Adult female | 0 / 5 | _ |
|  | Adult male | 0 / 1 | – |
| Not identified  (n = 15) | Adult female | 5 / 13 | *Candidatus* Midichloria mitochondrii  (n = 4)  *Francisella*-like endosymbiont (n = 1) |
|  | Nymph | 0 / 1 | – |
|  | Not identified | 0 / 1 | – |
| Total  (n = 165) | Adult female | 46 / 108  (42.6 %) | *Francisella*-like endosymbionts (n = 4)  *Coxiella* endosymbiont (n = 1)  *Candidatus* midichloria mitochondrii  (n = 39)  *Wolbachia pipientis* (n = 1)  *Candidatus* Trichorickettsia mobilis  (n = 1) |
|  | Adult male | 3 / 43  (7 %) | *Francisella*-like endosymbiont (n = 1)  *Candidatus* midichloria mitochondrii  (n = 2) |
|  | Nymph | 1 / 11 (9%) | *Coxiella* endosymbiont (n = 1) |

## Supplementary Table 4. Details of sequences deposited in GenBank for tick-borne pathogens and endosymbionts detected in dogs and their ticks.

| Organism | Sample ID | Geographic Location | Collection date | Target gene for sequencing | Isolation source (host) | Genbank accession number |
| --- | --- | --- | --- | --- | --- | --- |
| *Babesia canis canis* | C-23-4 | France: 63200 Mozac | 2023-02 | 18S ribosomal RNA | Dog blood (female) | PX092669 |
| *Babesia canis canis* | C-23-138 | France: 95300 Pontoise | 2023-09 | 18S ribosomal RNA | Dog blood (female) | PX092670 |
| *Babesia canis canis* | C-23-141 | France: 07410 Saint-Felicien | 2023-10 | 18S ribosomal RNA | Dog blood (male) | PX092671 |
| *Babesia canis canis* | C-23-142 | France: 37700 Saint-Pierre-des-Corps | 2023-10 | 18S ribosomal RNA | Dog blood (female) | PX092672 |
| *Babesia canis canis* | C-24-1 | France: 64130 Mauleon | 2023-03 | 18S ribosomal RNA | Dog blood (female) | PX092673 |
| *Babesia canis canis* | T-22-17 | France: 89000 Auxerre | 2022-11 | 18S ribosomal RNA | *Dermacentor reticulatus* (eng female) | PX092674 |
| *Babesia canis canis* | T-22-26 | France: 63200 Mozac | 2022-10 | 18S ribosomal RNA | *Dermacentor reticulatus* (non-eng male) | PX092675 |
| *Babesia canis canis* | T-23-79 | France: 39400 Morez | 2023-07 | 18S ribosomal RNA | Ixodid tick (semi-eng female) | PX092676 |
| *Babesia canis canis* | T-23-154 | France: 19340 Eygurande | 2023-09 | 18S ribosomal RNA | *Ixodes* spp. (eng female) | PX092677 |
| *Babesia canis canis* | T-23-167 | France: 37700 Saint-Pierre-des-Corps | 2023-10 | 18S ribosomal RNA | *Dermacentor reticulatus* (en female) | PX092678 |
| *Babesia canis canis* | T-23-176 | France: 07410 Saint-Felicien | 2023-11 | 18S ribosomal RNA | *Ixodes ricinus* (eng female) | PX092679 |
| *Babesia* sp. *venatorum* | T-23-66 | France: 12100 Millau | 2023-06 | 18S ribosomal RNA | *Ixodes ricinus* (semi-eng female) | PX092721 |
| *Babesia* sp. *venatorum* | T-23-86 | France: 32000 Auch | 2023-07 | 18S ribosomal RNA | *Ixodes* spp*.* (semi-eng female) | PX092722 |
| *Babesia* sp. *venatorum* | T-23-87 | France: 36700 Chatillon-sur-Indre | 2023-07 | 18S ribosomal RNA | *Ixodes ricinus* (semi-eng female) | PX092723 |
| *Borrelia garinii* | C-23-2 | France: 13118 Entressen | 2023-04 | 5S-23S intergenic spacer | Dog blood (male) | PX098505 |
| *Borrelia garinii* | C-23-115 | France: 49300 Cholet | 2023-07 | 5S-23S intergenic spacer | Dog blood (female) | PX098506 |
| *Borrelia garinii* | C-23-138 | France: 95300 PONTOISE | 2023-09 | 5S-23S intergenic spacer | Dog blood (female) | PX098507 |
| *Borrelia garinii* | C-23-141 | France: 07410 Saint-Felicien | 2023-10 | 5S-23S intergenic spacer | Dog blood (male) | PX098508 |
| *Borrelia garinii* | C-24-2 | France: 35170 BRUZ | 2023-03 | 5S-23S intergenic spacer | Dog blood (female) | PX098509 |
| *Borrelia garinii* | T-23-66 | France: 12100 Millau | 2023-06 | 5S-23S intergenic spacer | *Ixodes ricinus* (semi-eng female) | PX098510 |
| *Borrelia garinii* | T-23-89 | France: 46100 Figeac | 2023-07 | 5S-23S intergenic spacer | *Ixodes ricinus* (semi-eng female) | PX098511 |
| *Borrelia garinii* | T-23-159 | France: 54200 Toul | 2023-09 | 5S-23S intergenic spacer | *Dermacentor reticulatus* (non-eng male) | PX098512 |
| *Borrelia garinii* | T-23-161 | France: 95300 Pontoise | 2023-10 | 5S-23S intergenic spacer | *Dermacentor reticulatus* (eng female) | PX098513 |
| *Borrelia garinii* | T-23-174 | France: 07410 Saint-Felicien | 2023-10 | 5S-23S intergenic spacer | *Ixodes ricinus* (eng female) | PX098514 |
| *Borrelia garinii* | T-24-5 | France: 35170 Bruz | 2023-03 | 5S-23S intergenic spacer | *Dermacentor* spp.  (eng ticks) | PX098515 |
| *Wolbachia* sp. | C-23-56 | France: 76390 Aumale | 2023-05 | 16S ribosomal RNA | Dog blood (female) | PX120095 |
| *Candidatus* Midichloria mitochondrii | C-22-41 | France: 89000 Auxerre | 2022-11 | 16S ribosomal RNA | Dog blood (male) | PX103721 |
| *Candidatus* Midichloria mitochondrii | T-22-28 | France: 77700 Romainvilliers | 2022-10 | 16S ribosomal RNA | *Dermacentor reticulatus* (non-eng male) | PX103722 |
| *Candidatus* Midichloria mitochondrii | T-22-34 | France: 63200 Mozac | 2022-10 | 16S ribosomal RNA | *Dermacentor reticulatus* (semi-eng female) | PX103723 |
| *Candidatus* Midichloria mitochondrii | T-23-29 | France: 59770 Marly | 2023-05 | 16S ribosomal RNA | Non identified tick (semi-eng female) | PX103724 |
| *Candidatus* Midichloria mitochondrii | T-23-31 | France: 75020 Paris | 2023-05 | 16S ribosomal RNA | *Dermacentor reticulatus* (semi-eng female) | PX103725 |
| *Candidatus* Midichloria mitochondrii | T-23-32 | France: 76390 Aumale | 2023-05 | 16S ribosomal RNA | Non identified tick (semi-eng female) | PX103726 |
| *Candidatus* Midichloria mitochondrii | T-23-52 | France: 35220 Rennes | 2023-05 | 16S ribosomal RNA | *Ixodes ricinus* (semi-eng female) | PX103727 |
| *Candidatus* Midichloria mitochondrii | T-23-61 | France: 90300 Valdoie | 2023-06 | 16S ribosomal RNA | *Ixodes* spp. (semi-eng female) | PX103728 |
| *Candidatus* Midichloria mitochondrii | T-23-66 | France: 12100 Millau | 2023-06 | 16S ribosomal RNA | *Ixodes ricinus* (semi-eng female) | PX103729 |
| *Candidatus* Midichloria mitochondrii | T-23-69 | France: 95540 Mery-sur-Oise | 2023-06 | 16S ribosomal RNA | *Ixodes* spp. (semi-eng female) | PX103730 |
| *Candidatus* Midichloria mitochondrii | T-23-71 | France: 42190 Saint-Nizier-sous-Charlieu | 2023-06 | 16S ribosomal RNA | Non-identified tick (semi-eng female) | PX103731 |
| *Candidatus* Midichloria mitochondrii | T-23-72 | France: 95250 Beauchamp | 2023-06 | 16S ribosomal RNA | *Ixodes* spp. (semi-eng female) | PX103732 |
| *Candidatus* Midichloria mitochondrii | T-23-73 | France: 21000 Dijon | 2023-06 | 16S ribosomal RNA | *Ixodes* spp. (semi-eng female) | PX103733 |
| *Candidatus* Midichloria mitochondrii | T-23-74 | France: 74140 Saint Cergues | 2023-07 | 16S ribosomal RNA | *Ixodes ricinus* (semi-eng female) | PX103734 |
| *Candidatus* Midichloria mitochondrii | T-23-76 | France: 65400 Argeles Gazost | 2023-07 | 16S ribosomal RNA | *Ixodes ricinus* (semi-eng female) | PX103735 |
| *Candidatus* Midichloria mitochondrii | T-23-83 | France: 12100 Millau | 2023-07 | 16S ribosomal RNA | *Ixodes ricinus* (semi-eng female) | PX103736 |
| *Candidatus* Midichloria mitochondrii | T-23-86 | France: 32000 Auch | 2023-07 | 16S ribosomal RNA | *Ixodes* spp. (semi-eng female) | PX103737 |
| *Candidatus* Midichloria mitochondrii | T-23-88 | France: 46100 Figeac | 2023-07 | 16S ribosomal RNA | *Ixodes ricinus* (semi-eng female) | PX103738 |
| *Candidatus* Midichloria mitochondrii | T-23-92 | France: 12300 Degazeville | 2023-06 | 16S ribosomal RNA | *Ixodes ricinus* (semi-eng female) | PX103739 |
| *Candidatus* Midichloria mitochondrii | T-23-93 | France: 12300 Degazeville | 2023-06 | 16S ribosomal RNA | *Ixodes ricinus* (semi-eng female) | PX103740 |
| *Candidatus* Midichloria mitochondrii | T-23-154 | France: 19340 Eygurande | 2023-09 | 16S ribosomal RNA | *Ixodes* spp. (eng female) | PX103741 |
| *Candidatus* Midichloria mitochondrii | T-23-155 | France: 19340 Eygurande | 2023-09 | 16S ribosomal RNA | *Ixodes* spp. (eng female) | PX103742 |
| *Candidatus* Midichloria mitochondrii | T-23-156 | France: 19340 Eygurande | 2023-09 | 16S ribosomal RNA | *Ixodes* spp. (eng female) | PX103743 |
| *Candidatus* Midichloria mitochondrii | T-23-157 | France: 19340 Eygurande | 2023-09 | 16S ribosomal RNA | *Ixodes* spp (eng female) | PX103744 |
| *Candidatus* Midichloria mitochondrii | T-23-158 | France: 19340 Eygurande | 2023-09 | 16S ribosomal RNA | *Ixodes* spp. (eng female) | PX103745 |
| *Candidatus* Midichloria mitochondrii | T-23-163 | France: 07410 Saint-Felicien | 2023-10 | 16S ribosomal RNA | *Ixodes ricinus* (eng female) | PX103746 |
| *Candidatus* Midichloria mitochondrii | T-23-168 | France: 19340 Eygurande | 2023-10 | 16S ribosomal RNA | *Ixodes ricinus* (eng female) | PX103747 |
| *Candidatus* Midichloria mitochondrii | T-23-174 | France: 07410 Saint-Felicien | 2023-10 | 16S ribosomal RNA | *Ixodes ricinus* (eng female) | PX103748 |
| *Candidatus* Midichloria mitochondrii | T-23-178 | France: 47140 Saint-Sylvestre-sur-Lot | 2023-11 | 16S ribosomal RNA | *Ixodes ricinus* (eng female) | PX103749 |
| *Candidatus* Midichloria mitochondrii | T-23-181 | France: 46100 Figeac | 2023-11 | 16S ribosomal RNA | *Ixodes ricinus* (eng female) | PX103750 |
| *Candidatus* Midichloria mitochondrii | T-23-185 | France: 32000 Auch | 2023-11 | 16S ribosomal RNA | *Ixodes ricinus* (semi-eng female) | PX103751 |
| *Candidatus* Trichorickettsia mobilis | T-23-13 | France: 13118 Entressen | 2023-04 | 16S ribosomal RNA | *Rhipiceplalus sanguineus* (semi-eng female) | PX105156 |
| *Coxiella-like* endosymbiont | T-23-49 | France: 130090 Aix-en-Provence | 2023-05 | 16S ribosomal RNA | *Rhipiceplalus sanguineus* (female) | PX105157 |
| *Francisella*-like endosymbiont | T-22-2 | France: 95540 Mery-sur-Oise | 2022-11 | 16S ribosomal RNA | Non-identified tick (eng female) | PX103799 |
| *Francisella*-like endosymbiont | T-22-15 | France: 57290 Seremange | 2022-10 | 16S ribosomal RNA | *Dermacentor reticulatus* (non-eng female) | PX103800 |
| *Francisella*-like endosymbiont | T-23-183 | France: 94300 Vincennes | 2023-11 | 16S ribosomal RNA | *Dermacentor reticulatus* (semi-eng female) | PX103801 |
| *Rickettsia raoultii* | T-23-23 | France: 03330 Bellenaves | 2023-04 | 16S ribosomal RNA | *Dermacentor reticulatus* (non-eng male) | PX105158 |
| *Rickettsia massiliae* | T-23-11 | France: 13118 Entressen | 2023-04 | 16S ribosomal RNA | *Rhipiceplalus sanguineus* (semi-eng female) | PX103851 |
| *Rickettsia massiliae* | T-23-12 | France: 13118 Entressen | 2023-04 | 16S ribosomal RNA | *Rhipiceplalus sanguineus* (semi-eng female) | PX103852 |
| *Rickettsia massiliae* | T-23-14 | France: 13118 Entressen | 2023-04 | 16S ribosomal RNA | *Rhipiceplalus sanguineus* (semi-eng female) | PX103853 |
| *Rickettsia massiliae* | T-23-26 | France: 130090 Aix-en-Provence | 2023-04 | 16S ribosomal RNA | *Rhipiceplalus* spp. (semi-eng female) | PX103854 |
| *Rickettsia massiliae* | T-23-27 | France: 130090 Aix-en-Provence | 2023-04 | 16S ribosomal RNA | *Rhipiceplalus* spp. (semi-eng female) | PX103855 |
| *Rickettsia massiliae* | T-23-48 | France: 130090 Aix-en-Provence | 2023-05 | 16S ribosomal RNA | *Rhipiceplalus sanguineus*  (eng female) | PX103856 |
| *Rickettsia massiliae* | T-23-50 | France: 130090 Aix-en-Provence | 2023-05 | 16S ribosomal RNA | *Rhipiceplalus sanguineus* (non-eng female) | PX103857 |

## eng: engorged ; semi-eng: semi-engorged; non-eng: non-engorged.

## Supplementary Table 5. Other microorganisms (microbiota) detected in ticks removed from dogs, according to tick species and developmental stage.

| Tick species (total) | Developmental stage | PCR-positive /tested | | Detected microbiota (n) |
| --- | --- | --- | --- | --- |
| *Dermacentor reticulatus*  (n = 49) | Adult female | 6 / 23 | *Agrobacterium* spp. + *Conexibacter* spp. (1)  *Bacillus paranthracis* (1)  *Conexibacter* spp. (1)  *Hydrocarboniphaga* spp. (1)  Uncultured bacterium (1)  Uncultured clostridial (1) | |
|  | Adult male | 9 / 26 | *Conexibacter* spp. (1)  *Serratia marcescens* (1)  Uncultured bacterium (4)  Uncultured *Phenylobacterium* (1)  Uncultured bacterium + *Conexibacter* spp. (1)  Uncultured *Serratia* spp. (1) | |
| *Dermacentor marginatus*  (n = 1) | Adult female | 0 / 1 |  | |
| *Dermacentor* spp.  (n = 14) | Adult female | 2 / 11 | *Conexibacter* spp. (1)  *Serratia marcescens* (1) | |
|  | Adult male | 1 / 1 | *Bacillus thuringiensis* (1) | |
|  | Not identified | 0 / 2 |  | |
| *Ixodes hexagonus*  (n = 11) | Nymph | 6 / 10 | *Enterococcus faecalis* (1)  *Phenylobacterium* spp. (1)  Uncultured bacterium (4) | |
| *Ixodes ricinus*  (n = 30) | Adult female | 4 / 26 | *Bacillus toyonensis* (1)  *Bacillus thuringiensis* (1)  *Enterococcus* spp. + *Conexibacter* spp. (1)  Uncultured bacterium (1) | |
|  | Adult male | 0 / 4 | / | |
| *Ixodes* spp.  (n = 12) | Adult female | 0 / 12 | / | |
| *Rhipicephalus sanguineus* s.l.  (n = 27) | Adult female | 0 / 16 | / | |
|  | Adult male | 0 / 11 | / | |
| *Rhipicephalus* spp.  (n = 6) | Adult female | 0 / 5 | / | |
|  | Adult male | 0 / 1 | / | |
| Not identified  (n = 15) | Adult female | 1 / 13 | *Serratia marcescens* (1) | |
|  | Nymph | 0 / 1 | / | |
|  | Not identified | 0 / 1 | / | |
| Total  (n = 165) | Adult female | 13 / 108  (12 %) | *Agrobacterium* spp. + *Conexibacter* spp. (1)  *Bacillus paranthracis* strain KA1 (1)  *Bacillus toyonensis* (1)  *Bacillus thuringiensis* (1)  *Conexibacter* spp. (2)  *Enterococcus* spp. + *Conexibacter* spp. (1)  *Hydrocarboniphaga* spp. (1)  *Serratia marcescens* (2)  Uncultured bacterium (2)  Uncultured clostridial (1) | |
|  | Adult male | 10 / 43  (23.3 %) | *Bacillus thuringiensis* (1)  *Conexibacter* spp. (1)  *Serratia marcescens* (1)  Uncultured bacterium (4)  Uncultured bacterium + *Conexibacter* spp. (1)  Uncultured *Phenylobacterium* (1)  Uncultured *Serratia* spp. (1) | |
|  | Nymph | 6 / 11 (54.5 %) | *Enterococcus faecalis* (1)  *Phenylobacterium* spp. (1)  Uncultured bacterium (4) | |
|  | Not identified | 0 / 3 | / | |
